# RETRACTION: Influences of Ultrasound Osteotome on Wound Infection and Wound Complications Following Removal of Mandibular Wisdom Teeth

**DOI:** 10.1111/iwj.70170

**Published:** 2024-12-15

## Abstract

**RETRACTION:** LiQ. , LiuD. , WeiX. , LiZ. , WenM. , HouZ. , and ZhangW. , “Influences of Ultrasound Osteotome on Wound Infection and Wound Complications Following Removal of Mandibular Wisdom Teeth,” International Wound Journal 21, no. 1 (2024): e14618, 10.1111/iwj.14618.38272826 PMC10789503

The above article, published online on 15 January 2024, in Wiley Online Library (http://onlinelibrary.wiley.com/), has been retracted by agreement between the journal Editor in Chief, Professor Keith Harding; and John Wiley & Sons Ltd. Following an investigation by the publisher, all parties have concluded that this article was accepted solely on the basis of a compromised peer review process. The editors have therefore decided to retract the article. The authors did not respond to our notice regarding the retraction.

**RETRACTION:** Q. Li , D. Liu , X. Wei , Z. Li , M. Wen , Z. Hou , and W. Zhang , “Influences of Ultrasound Osteotome on Wound Infection and Wound Complications Following Removal of Mandibular Wisdom Teeth,” International Wound Journal 21, no. 1 (2024): e14618, 10.1111/iwj.14618.38272826 PMC10789503

The above article, published online on 15 January 2024, in Wiley Online Library (http://onlinelibrary.wiley.com/), has been retracted by agreement between the journal Editor in Chief, Professor Keith Harding; and John Wiley & Sons Ltd. Following an investigation by the publisher, all parties have concluded that this article was accepted solely on the basis of a compromised peer review process. The editors have therefore decided to retract the article. The authors did not respond to our notice regarding the retraction.

